# Information Decomposition of Target Effects from Multi-Source Interactions: Perspectives on Previous, Current and Future Work

**DOI:** 10.3390/e20040307

**Published:** 2018-04-23

**Authors:** Joseph T. Lizier, Nils Bertschinger, Jürgen Jost, Michael Wibral

**Affiliations:** 1 Complex Systems Research Group and Centre for Complex Systems, Faculty of Engineering & IT, The University of Sydney, NSW 2006, Australia; 2 Frankfurt Institute of Advanced Studies (FIAS) and Goethe University, 60438 Frankfurt am Main, Germany; 3 Max Planck Institute for Mathematics in the Sciences, Inselstraße 22, 04103 Leipzig, Germany; 4 Santa Fe Institute, 1399 Hyde Park Road, Santa Fe, NM 87501, USA; 5 MEG Unit, Brain Imaging Center, Goethe University, 60528 Frankfurt, Germany; 6 Max Planck Institute for Dynamics and Self-Organization, 37077 Göttingen, Germany

**Keywords:** mutual information, information decomposition, unique information, redundant information, complementary information, redundancy, synergy, 89.70.Cf, 89.75.Fb, 05.65.+b, 87.19.lo

## Abstract

The formulation of the Partial Information Decomposition (PID) framework by Williams and Beer in 2010 attracted a significant amount of attention to the problem of defining redundant (or shared), unique and synergistic (or complementary) components of mutual information that a set of source variables provides about a target. This attention resulted in a number of measures proposed to capture these concepts, theoretical investigations into such measures, and applications to empirical data (in particular to datasets from neuroscience). In this Special Issue on “Information Decomposition of Target Effects from Multi-Source Interactions” at Entropy, we have gathered current work on such information decomposition approaches from many of the leading research groups in the field. We begin our editorial by providing the reader with a review of previous information decomposition research, including an overview of the variety of measures proposed, how they have been interpreted and applied to empirical investigations. We then introduce the articles included in the special issue one by one, providing a similar categorisation of these articles into: i. proposals of new measures; ii. theoretical investigations into properties and interpretations of such approaches, and iii. applications of these measures in empirical studies. We finish by providing an outlook on the future of the field.

## 1. Background to Information Decomposition

Shannon information theory [1,2,3] has provided rigorous ways to capture our intuitive notions regarding uncertainty and information, and it has made an enormous impact in doing so. One of the fundamental measures here is mutual information I(S;T), which captures the average information contained in samples *s* of a set of source variables *S* about samples *t* of another variable *T*, and vice versa. If we have two source variables $S_{1},S_{2}$ and a target *T*, for example, we can measure:

1. the information held by one source about the target $I(S_{1};T)$,
2. the information held by the other source about the target $I(S_{2};T)$, and
3. the information jointly held by those sources together about the target $I(\left\{S_{1},S_{2}\right\};T)$.

Any other notion about the directed information relationship between these variables which can be captured by classical information-theoretic measures (e.g., conditional mutual information terms $I(S_{1};T|S_{2})$ and $I(S_{2};T|S_{1})$) is redundant with those three quantities.

However, intuitively, there is a strong desire to measure further notions of how this directed information interaction may be decomposed, e.g., for these two sources:

1. how much *redundant* or *shared* information $R(S_{1},S_{2}\;\to \;T)$ the two source variables hold about the target,
2. how much *unique* information $U(S_{1}\;\setminus \;S_{2}\;\to \;T)$ source variable $S_{1}$ holds about *T* that $S_{2}$ does not,
3. how much *unique* information $U(S_{2}\;\setminus \;S_{1}\;\to \;T)$ source variable $S_{2}$ holds about *T* that $S_{1}$ does not, and
4. how much *complementary* or *synergistic* information $C(S_{1},S_{2}\;\to \;T)$ can only be discerned by examining the two sources together.

These notions go beyond the traditional information-theoretic view of a channel serving the purpose of reliable communication, considering now the situation of multiple communication streams converging on a single target. This is a common situation in biology, and in particular in neuroscience, where, say, the ability of a target to synergistically fuse multiple information sources in a non-trivial fashion is likely to have its own intrinsic value, independently of reliability of communication.

**Figure 1:**
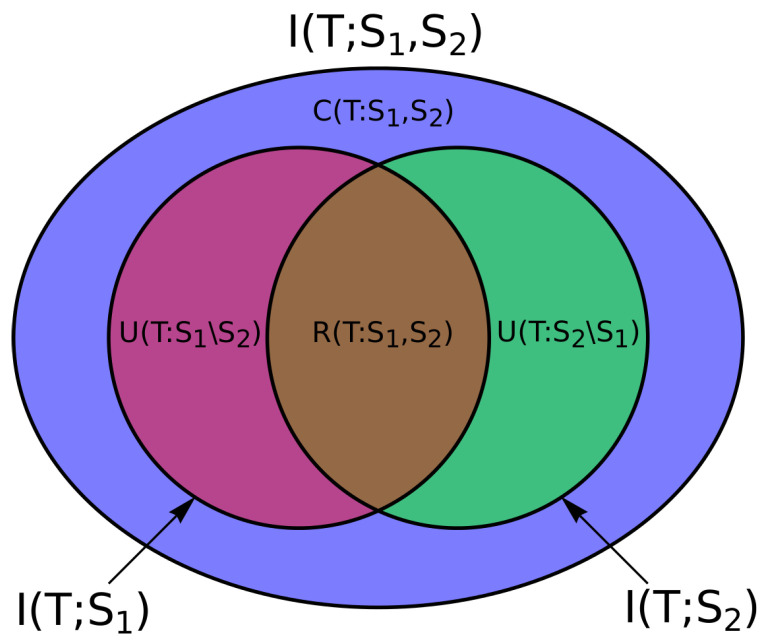
Partial information diagram for two sources to a target showing the relationship of the partial information quantities to the fundamental mutual information terms.

The absence of (completely satisfactory) measures for such decompositions into redundant, unique and synergistic information has arguably been the most fundamental missing piece in classical information theory. Contemporary work on this problem was triggered by the formulation of the Partial Information Decomposition (PID) framework in a landmark paper by Williams and Beer [4] in 2010 (*note*: this paper was refined under an alternate title, and circulated privately only as [5]). This framework suggested that these quantities were related to the fundamental mutual information measures as follows and shown in Figure 1 for two source variables (with more complex relations for higher order interactions):

(1) $$\begin{array}{cc} I(\left\{S_{1},S_{2}\right\};T) & =R(S_{1},S_{2}\;\to \;T)+U(S_{1}\;\setminus \;S_{2}\;\to \;T)+U(S_{2}\;\setminus \;S_{1}\;\to \;T)+C(S_{1},S_{2}\;\to \;T), \end{array}$$

(2) $$\begin{array}{cc} I(S_{1};T) & =R(S_{1},S_{2}\;\to \;T)+U(S_{1}\;\setminus \;S_{2}\;\to \;T), \end{array}$$

(3) $$\begin{array}{cc} I(S_{2};T) & =R(S_{1},S_{2}\;\to \;T)+U(S_{2}\;\setminus \;S_{1}\;\to \;T). \end{array}$$

Crucially, the PID framework proposed that all these components coexist, subverting what had come to be the established interpretation [6] of the interaction information $II=I\left(S_{1};T|S_{2}\right)-I\left(S_{1};T\right)$, that II>0 implied a synergistic interaction whilst II<0 implied redundancy (and implying them to be mutually exclusive). Indeed, the PID framework revealed II as a net of synergy and redundancy terms (i.e., net synergy). Crucially, the PID framework proposed a set of axioms—*symmetry*, *self-redundancy* and *monotonicity*—that a measure of redundancy (for an arbitrary number of source variables to a target) should satisfy [5] (see summary e.g., in [7]). While these axioms were not sufficient to uniquely lock in a measure of redundancy, they do specify a partial ordering for redundancy terms across various joint collections of sources, and an algebra for how to compute *partial information atoms* attributed to such collections of sources (but no simpler collection) at nodes in a *partial information lattice* representing the hierarchy according to this ordering. This approach proved particularly appealing to the community.

In that paper, Williams and Beer [4] also proposed one measure of redundancy that satisfied the axioms they had laid out, known as $I_{\min}$. This measure found less favour in the community than the framework itself, encountering various criticisms such as that it did not distinguish “the *same* information or just the *same amount* of information” [8] (see also [7,9,10]), and did not satisfy a chain rule across multiple target variables [8]. However, perhaps the most controversy surrounded interpretation of the *Two-bit-copy* example (where a target is a copy of two IID input bits), which $I_{\min}$ suggested to be 1 bit redundant and 1 bit synergistic information, yet other authors felt should be 1 bit of unique information from each source because “the wires don’t even touch” [10], p. 167. Indeed, the strong intuition some felt on this interpretation led Harder et al. [9] to suggest a 4th axiom (known as *identity*) requiring the redundancy in such copying situations to be equal to the mutual information between the two source variables.

Following these developments, the past few years witnessed a concentration of work by the community in proposing, contrasting, and investigating new measures to capture these notions of information decomposition. (See an earlier review by Wibral et al. [11], in Section 4 of that article). Primarily amongst these were the information-geometry based $I_{\mathrm{red}}$ from Harder et al. [9], and $S_{\mathrm{VK}}$ from Griffith and Koch [10] and $\tilde{UI}$ from Bertschinger et al. [12], all of which were presented only for a pair of sources. The latter two approaches were later found to be equivalent, and attracted much attention due to being placed on a particularly rigorous mathematical footing, despite computational difficulties in solving the convex optimisation they require. For example, the derivation of the measure by Bertschinger et al. [12] followed directly (rather than being posed ad-hoc) from an assumption that existence of unique information depended only on the pairwise marginal distributions between the individual sources and the target (known as “Assumption (*)”). Furthermore, the measure was given an operational interpretation in terms of how unique information could be exploited in decision problems. Finally, many mathematical properties of the approach were proven by Bertschinger et al. [12] and in follow up papers by these authors [13,14].

Yet while many authors welcomed the new measures for satisfying the identity property, it was quickly realised that they did not completely solve the search for a redundancy measure for an arbitrary number of variables. This is because Rauh et al. [13] demonstrated that no redundancy measure can satisfy the identity property along with the original axioms of Williams and Beer [4] and still provide non-negative partial information atoms when we have more than two source variables.

As a consequence, the search for candidate redundancy measures continued, with various groups considering to drop either the identity property or one or more of the original Williams and Beer [4] axioms. Olbrich et al. [14] and Perrone and Ay [15] investigated the possibility of defining synergy via projections of probability distributions to those retaining only certain orders of interactions (in particular using exponential families), while Rosas et al. [16] sought similar decompositions for joint entropies. Some approaches sought to construct intermediate variables that could be used to represent components of the decomposition, e.g., the investigation of Gács-Körner common information by Griffith et al. [17], Griffith and Ho [18] and constructions of variables to contain synergy only by Quax et al. [19]. Others investigated relatively simpler mechanisms such as the minimum mutual information (MMI) provided by any source by Barrett [20] (and the related approach by Chatterjee and Pal [21]).

Meanwhile, other theoretical developments were taking place in parallel. One line of work considered how these measures relate to concepts of distributed information processing in terms of information storage, transfer and modification [7,22,23,24]. Lizier et al. [7] made a case that information decomposition approaches should (at least) be interpretable on pointwise or event-wise realisations of the source and target variables, rather than only with their averages. Barrett [20] began considering continuous-valued variables, and indeed showed that the minimum mutual information was a unique form of the redundancy for linearly coupled Gaussian variables, for two sources, under the Williams and Beer [4] axioms and Bertschinger et al.’s [12] Assumption (*). Others provided detailed comparisons between the measures and catalogued results from various logic gates (e.g., [25]).

Despite the lingering issues surrounding a definitive measure of redundancy, the desire for using such measures has been intense, and applications have been made drawing on the variety of measures listed above. Computational neuroscience in particular emerged as a primary application area due to significant interest in questions surrounding how target neurons integrate information from large numbers of sources, as well as the availability of data sets on which to investigate these questions. For example, Timme et al. [25] contrasted $I_{\min}$ with several earlier candidates regarding the decomposition of information contributions between various electrode measurements from developing neural cultures, concentrating in particular on how redundancy and synergy generally increase during development. Later, Timme et al. [24] applied the PID view of information modification of Lizier et al. [7] to study dynamics of spiking activity of neural cultures incorporating history vectors of the target neuron, finding that neurons which modify “large amounts of information tended to receive connections from high out-degree neurons” in the effective network structure. Stramaglia et al. [26] use interaction information or net synergy interpretations to study interactions in electroencephalography (EEG) measurements in pre-seizure states for an epileptic patient. Further, Wibral et al. [27] applied PID to make various, theoretically proposed neural goal functions–such as infomax [28]–comparable, and were able to clarify whether the theories do indeed represent the information components that they had aimed at. Applications also began to emerge in examinations of biological data sets (e.g., [21,29]), and in gambling [30].

## 2. Contents of the Special Issue

In December 2016 we held an informal workshop on Partial Information Decomposition at the Frankfurt Institute for Advanced Studies and the Goethe University, bringing together some of the leading research groups in the field to discuss their latest developments. The workshop revealed a strong level of new activity in the area, and triggered deep discussions in particular regarding how further progress towards a measure may be made and which axiom(s) may need to be dropped/changed for this to occur. The attendees expressed a desire for publications of such new activity to be gathered in a common location, resulting in this Special Issue. The issue seeks to bring together the new efforts presented at the workshop, to capture a snapshot of current research, as well as to provide impetus for and focused scrutiny on newer work. We also seek to present progress to the wider community and attract further research in this area. In scope for the issue were research articles proposing new measures or pointing out future directions, review articles on existing approaches, commentary on properties and limitations of such approaches, philosophical contributions on how such measures may be used or interpreted, applications to empirical data (e.g., neural data), and more.

The contributions we have published can be classified under three key themes: new PID measures, theoretical investigations (including examinations of numerical estimators), and applications.

### 2.1. New Measures of Redundancy

Considering the first, perhaps not-so surprising theme, our Special Issue carries three papers proposing new measures of redundancy.

Rauh et al. [31] present the *extractable shared information* as a redundancy measure for the bivariate case. The key feature of this measure is that, in contrast to previous proposals, it satisfies the property of target or left monotonicity (i.e., that the redundancy is non-decreasing when more target variables are added [8], or restated here as redundancy being non-increasing when a new target variable is a function of the old target). This is achieved via a construction which translates any measure of shared information into one that satisfies this property. The authors then explore the properties of this measure, and show for example that it is not compatible with a Blackwell interpretation of unique information (see their other contribution, [32], discussed in Section 2.2).

Ince [33] constructs a measure $I_{CCS}$ of redundancy by directly examining common values of pointwise mutual information (or change in surprisal) in each realisation of the variables. Interestingly, Ince [33] considers positive and negative pointwise information as fundamentally different and treats their occurrence separately, counting redundancy only from pointwise co-information terms when the signs of all relevant change in surprisal terms align. This necessitates considering redundant misinformation as well as redundant information (and related terms such as unique misinformation). The author argues for the justification of these new perspectives as well other properties of the measure, including replacing a requirement of monotonicity with subset equality (which had usually been considered only as part of monotonicity) and the use of a modified independent identity axiom introduced here. Ince [33] also provides a game-theoretic operational interpretation to argue for the approach presented, contrasting this with the decision-theoretic operational interpretation from Bertschinger et al. [12]. This line of work continues in a companion paper [34].

From a similar pointwise perspective, Finn and Lizier [35] build on earlier work to now directly identify positive and negative components of pointwise information from each source to the target as *specificity* and *ambiguity* [36], and argue that redundancies in these should be treated independently to avoid blurring them (in the same way that PID originally sought to avoid how interaction information blurs synergy and redundancy). The authors introduce a new example called “Pointwise Unique”, where in any pointwise configuration only one source holds non-zero information about the target. They demonstrate that other existing measures do not identify unique information in this case, unlike their new approach. They also introduce a new operational interpretation of redundancy in terms of probability mass diagrams, and in allowing negative terms in net, show that their pointwise and component-wise approach is unique in satisfying a chain-rule over target variables. The latter feature also allows the approach to provide a consistent answer to *Two-bit-copy* of 1 bit redundant and 1 bit synergistic information, regardless of the order in which target bits are decomposed.

It is interesting to note that the latter two of these new approaches independently make similar departures from the status quo here: both taking a “bottom-up” pointwise information perspective, considering negative partial information terms, dropping the identity axiom, and being extendible to three or more source variables.

### 2.2. Theoretical Investigations

Next, the special issue contains a number of theoretical investigations into the properties of PID approaches in general and with regard to specific measures.

James and Crutchfield [37] make the case for measures of information decomposition beyond the standard Shannon measures by seeking to differentiate two examples of three variable systems: one constructed with dyadic dependencies and the other with triadic. Via a comprehensive analysis, they show that no standard Shannon measure can differentiate between the two examples, whilst various measures of information decomposition, e.g., Gács-Körner common information and the Bertschinger et al. [12] PID, are able to. Whilst these two PID approaches do provide such a differentiation, the authors express a general desire for the additional existence of a symmetric decomposition that does not partition variables into sources and targets.

Pica et al. [38] examine a two-source one-target PID from three perspectives in total, i.e., one perspective for each variable as the target, in order to examine commonalities between the perspectives. Assuming non-negativity but not any specific PID measure, they identify only seven non-negative information subatoms that are required to construct each of the three PIDs in full, subject to knowing the ordering of the three redundancy terms. The authors also suggest novel definitions for a split between source redundancy (arising from correlations between the source variables) and non-source redundancy. Indeed, the authors use their approach to provide further insights into the information structure of the dyadic-vs-triadic example of James and Crutchfield [37].

Rauh [39] identifies the cryptographic interpretation of secret sharing as a useful model to consider information decomposition, since secret sharing schemes incorporate specific understanding of which subsets of participants have information about the secret. The author establishes correspondence between secret sharing and PID, and then uses this approach as a model to explore the partial information lattice. Negative terms in the lattice are identified for more than two participants (analogous to the argument by Rauh et al. [13]), which leads the author to discuss whether and how such terms could or should be interpreted, and subsequently questions whether the lattice needs to be extended or improved in some fashion.

Rauh et al. [32] examine the decision-theoretic Blackwell partial order, which ranks information channels (with a common input) according to the utility that can be obtained when decisions are made on the channel outputs. The authors present the unexpected result that a coarse-graining of one channel output may actually result in improved utility. They go on to compare the Blackwell ordering to mutual information, and discuss implications of the result for information decomposition.

Faes et al. [40] utilise vector autoregressive Gaussian models and the MMI measure, coupled with the aforementioned perspective of information modification, to examine the decomposition of contributions from information sources to a target over various temporal scales. The method of investigating the decomposition of contributions across different scales is achieved by a combination of filtering and then downsampling, and synthetic examples in the first instance are used to demonstrate that the method can reveal quite different decompositions at different temporal scales due to contrasting fast and slow dynamics. The authors then apply the approach to intracranial EEG data obtained prior to and during epileptic seizures, revealing in particular how synergistic and unique information transfer components change with scale.

Makkeh et al. [41] consider the the convex optimisation problem that must be solved in order to evaluate the Bertschinger et al. [12] approach, continuing on from the original observations by Bertschinger et al. [12] that Mathematica could not directly solve these optimisation problems. The authors provide both theoretical and practical perspectives, discussing various algorithmic approaches to the problem and why some perform poorly, and empirically comparing the performance of a number of software packages. Importantly, the authors identify two software packages which perform satisfactorily, and make recommendations regarding their use here.

### 2.3. Applications of Information Decomposition

Applications of PID form a substantial class of papers in our special issue. As identified above, neural applications (in addition to the EEG analysis by Faes et al. [40] above) account for the largest portion of these.

Kay et al. [42] consider the PID between a neural receptive field input and the signal modulating (amplifying or suppressing) it, giving rise to an output signal. In particular they demonstrate that, contrary to intuition from some perspectives, a modulatory signal can affect the transmission of information about other inputs without being transmitted itself. The authors go on to apply the Ince [33] and Bertschinger et al. [12] PID measures, as well as a related decomposition of entropy by Ince [34], to results from a visual contrast detection task in order to demonstrate that such forms of modulation may occur in real neural systems.

Wibral et al. [43] apply PID to decompose information storage, transfer and in particular information modification in developing neural cultures, following the perspective of Lizier et al. [7]. Utilising the Bertschinger et al. [12] PID measure via the publicly available IDT${}^{x}l$ toolkit [44], the authors identify the aforementioned components of information processing from pairs of input (multi-unit) spike train recordings to each output recording. They report that information modification initially rose during development with maturation of the culture (indicating intricate processing capabilities), followed by a decay when redundant information among neurons took over (possibly due to a lack of external inputs).

Moving on to artificial neural computation then, Tax et al. [45] also use PID to analyse neural development, but this time the development of a restricted Boltzmann machine during training. The authors focus on decomposing the information held by (sample pairs of) individual hidden neurons about the target variable to be classified, using $I_{\min}$ [4]. They observe a first phase where neurons appear to learn predominantly redundant information about the target, followed by a second phase where the neurons specialise to learn unique information about the target (also with a significant synergistic component). Further, the authors report that while larger networks appear to utilise higher order representations to a greater extent, individuals in smaller networks appear to learn more unique details, and conclude that perhaps network size pressure on learning can lead to disentangled representations.

Ghazi-Zahedi et al. [46] apply PID in order to further our understanding of morphological computation, “processes in the body that would otherwise have to be conducted by the brain”. Examining the embodied concept of the sensorimotor loop model, the authors quantify morphological computation as synergistic information from the cognitive system’s actuators and the current world state (incorporating both the system’s morphology and the part of the environment that can be affected by and affects the system) to the next world state. The authors focus on the synergy measure of Perrone and Ay [15] for this purpose, comparing it to previous measures and finding it to be generally more reliably oriented with their intuition, though not in all cases.

As highlighted above, computational biology has also emerged as an interesting application area for PID, and here Maity et al. [47] use PID to examine cross-talk in biochemical networks between two mitogen-activated protein kinase (MAPK) pathways. The authors examine data from models of these pathways, using Gaussian model calculations of the information-theoretic terms and quantifying net synergy. They demonstrate differences in information decomposition between different pathway architectures, e.g., signal integration motifs and signal bifurcation motifs.

Sootla et al. [48] turn our attention to various canonical complex systems, demonstrating how PID can provide still new insights into these well-understood examples. Utilising the Bertschinger et al. [12] PID (building on work by some of the authors on estimators for this measure in another contribution to the special issue [41]), the authors begin by examining decomposition of information in triplets of spins in the 2D Ising model, while the temperature is varied. They report that redundant information is maximised at the critical point, whilst synergistic information peaks in the disordered phase. Next, the authors decompose information of cells in 1D elementary cellular automata (ECA) from the two neighbouring sources of those cells. They perform a dimensionality reduction on the PID atoms (as dimensions), identifying some (but not perfect) distinction in characteristics between Wolfram’s rule classes.

## 3. Outlook

Information decomposition into redundant, unique and synergistic components has been recognised as a crucial theoretical problem which has proven far more difficult to solve than may have been expected. Thankfully, there is very strong activity in the community leading to progress on information decomposition approaches, which as outlined above is well reflected in this special issue. We hope that our presentation of these papers will further the debate regarding which is the “right” measure of redundancy, which original assumptions or axioms may need to be dropped or changed (as per new measures and challenges to current thinking in Section 2.1), and how the approaches can and should be interpreted and/or extended (as per investigations in Section 2.2). Certainly there is a hunger for applications of information decomposition (as per Section 2.3), and again we hope that the special issue helps to disseminate and encourage these approaches.
